# Prevalence and Severity of Depression, Anxiety, and Stress Among Tribal and Nontribal Adolescents in a Rural Block of Jharkhand: A Cross-Sectional Study

**DOI:** 10.7759/cureus.114694

**Published:** 2026-08-17

**Authors:** Rajdeep Mazumdar, Vidya Sagar, Anit Kujur, Shalini Sunderam

**Affiliations:** 1 Department of Community Medicine, Rajendra Institute of Medical Sciences, Ranchi, IND

**Keywords:** dass-21, depression prevalence, mental health disparities, rural jharkhand, tribal adolescent

## Abstract

Background: Adolescent mental health in India is an emerging health concern, particularly in the tribal population. Tribal populations are particularly vulnerable due to structural disadvantages arising from lower educational status, poverty, and limited health care access. While tribal communities make up a substantial proportion of Jharkhand’s population, epidemiological information on the mental health of tribal adolescents remains underrepresented.

Methods: Between December 2024 and June 2025, a cross-sectional comparative investigation was undertaken in the Rural Health Training Centre’s field area, Ormanjhi Block, Rajendra Institute of Medical Sciences, Ranchi. The enrolled study population comprised 310 adolescents aged 10-19 years, of whom 137 were tribal and 173 were nontribal. Assessment of socioeconomic status was carried out based on the modified B.G. Prasad Scale 2024, and depression, stress, and anxiety were evaluated using the Depression, Anxiety, and Stress Scale-21. Intergroup differences were examined using chi-square tests, Mann-Whitney U tests, and independent t-tests, with a p value of 0.05 considered statistically significant.

Results: Symptoms of stress and depression were each observed in 63.5% of the study participants, whereas anxiety was identified in 79.0%. Tribal adolescents experienced depression more frequently than their nontribal counterparts (71.5% vs. 57.2%; χ² = 6.76, p = 0.009, odds ratio = 1.88, 95% confidence interval: 1.16-3.03). The pattern of depression severity also varied between the two groups (χ² = 14.7, p = 0.005), with moderate-to-extremely severe depression affecting 59.1% of tribal adolescents compared with 39.8% of nontribal adolescents. In addition, the average depression score was significantly greater in tribal adolescents (15.5 ± 9.27 vs. 12.8 ± 9.61, p = 0.014).

Conclusions: Rural adolescents in Jharkhand bear a high mental health burden, with tribal adolescents disproportionately affected by depression. Structural determinants, including parental education and socioeconomic status, along with psychosocial factors such as social support, are important potential covariates. Mental health screening programs that are school-based and culturally acceptable interventions can address this mental health crisis.

## Introduction

The World Health Organization defines adolescence as the period between 10 and 19 years of age, representing a crucial developmental transition marked by rapid physical, cognitive, and psychosocial changes [1]. Experiencing depression, anxiety, and stress during these formative years not only affects social development and educational performance but also serves as a precursor to substance dependency, self-harming behaviors, and even suicide. From a global perspective, one in seven adolescents experiences a mental health disorder, with 50% of these arising in mid-adolescence [2]. The vulnerability is exacerbated by economic and social disparities, as the greatest burden of adolescent psychiatric disorders is faced by developing countries where mental-health infrastructure remains limited [3].

The National Mental Health Survey (NMHS 2015-2016) of India reported a 7.3% prevalence of psychiatric disorders among Indian adolescents aged 13-17 years [4,5]. This figure is arguably conservative as NMHS recorded only clinically diagnosed mental health disorders, leaving subclinical conditions unreported. Community-based studies in recent years reported prevalence rates ranging from 35% to 45% for depression and anxiety in Indian adolescents [6,7]. These burdens were again worsened by the COVID-19 pandemic, as documented by significant psychological distress among young people [8,9].

India’s tribal population accounts for 8.6% of the country’s population; Jharkhand is unique in that 26.2% of its population comprises Scheduled Tribes, according to the National Census 2011 [10]. These communities suffer from disadvantages like sociocultural marginalization, lower educational status, and higher poverty rates, but empirical evidence regarding mental health burden in tribal youth of Jharkhand remains scarce, and current literature heavily favors nontribal cohorts [11-14].

Adolescent mental health is identified as a public health priority, yet there remains a major gap in rural areas of Jharkhand. Existing studies frequently overlook these regions by omitting tribal cohorts or relying on diagnostic parameters or nonvalidated screening tools or focusing on urban populations [15,16]. Comparative analyses evaluating psychiatric disparities between tribal and nontribal adolescents living in the same geographical boundaries are limited, and gathering these baseline data is imperative for developing targeted, culturally tailored interventions and shaping effective state-level mental health policies.

To evaluate mental health outcomes, we administered the Depression, Anxiety, and Stress Scale-21 (DASS-21), a validated community-screening tool. In our study, the scale exhibited an internal consistency (Cronbach’s α = 0.93). DASS-21 has demonstrated high reliability (typically with a Cronbach’s α > 0.90) and construct validity across studies, including those involving adolescents in India [17-20].

To address these gaps, the present study aimed to 1) determine the prevalence and severity of stress, depression, and anxiety among rural adolescents in Ormanjhi Block, Ranchi, Jharkhand, and 2) compare these mental health outcomes between tribal and nontribal adolescents.

## Materials and methods

Study design and setting

A cross-sectional study was conducted in the rural field practice area under the Rural Health Training Centre (RHTC), Department of Community Medicine, Rajendra Institute of Medical Sciences (RIMS), Ormanjhi Block, Ranchi, Jharkhand. The estimated population of Ormanjhi Block is 94,137, representing a typical rural setting in Jharkhand, of which approximately 36% belong to Scheduled Tribes, as per Census 2011. The field practice area comprises four panchayats, namely Ormanjhi Bazartand, Irba, Chakla, and Anandi, with an estimated population of 25,500. There are 25 health subcenters, two primary health centers, and one community health center that cater to the health needs of the population; however, availability of mental health services is lacking at all levels of healthcare infrastructure in this area.

Study period

Data collection took place during the academic year 2024-2025. Data collection began in December 2024 and continued until June 2025. All participants were interviewed within the field practice area of RHTC, RIMS, Ormanjhi Block, Ranchi, Jharkhand.

Study population

The study involved adolescents aged 10-19 years who had resided in the field practice area for at least six months. Both tribal and nontribal adolescents were included for comparative analysis. Tribal status was identified on the basis of Article 342 of the Indian Constitution. Our study area comprised mainly the Oraon and Munda tribes, with a minor share of other tribal communities such as the Bedia and Santhals.

Operational Definition of Tribal Population

Participants were categorized as "tribal" if they self-identified as any of a recognized Scheduled Tribe community listed under Article 342 of the Constitution of India for the state of Jharkhand. The self-reported tribal status was cross-checked against the state's Scheduled Tribe roster using participants' family surnames.

Participants residing in the same area who were not Scheduled Tribes were categorized as "nontribals."

Sample size and sampling

Using the Cochran formula $n = \frac{Z^2 p(1-p)}{d^2}$ and assuming Z = 1.96 (for 95% confidence interval (CI)), anticipated prevalence p = 0.22 (Saikia et al. reported prevalence of mental health morbidities among school going Indian adolescents to be 22.2%) [7] and d = 0.05 (margin of error) baseline sample was calculated as 264. After adjusting for 15% nonresponse, the final sample size was 310. Calculations were verified using OpenEpi (Dean AG, Sullivan KM, Soe MM. OpenEpi: Open Source Epidemiologic Statistics for Public Health, Version. www.OpenEpi.com, updated 2013/04/06) [21].

A total of 340 adolescents were interviewed through consecutive sampling of households registered under RHTC. Out of these, 310 participants, comprising 137 tribal (44.2%) and 173 nontribal (55.8%) adolescents, responded, and their responses were deemed adequate for analysis. Thirty participants (8%) did not complete the interview. All eligible adolescents providing informed consent or assent were included.

Inclusion criteria

Inclusion

The study included adolescents aged 10-19 years, residing in the area for at least six months and willing to participate with informed consent from parents/guardians and assent from adolescents.

Exclusion

Adolescents with known psychiatric diagnoses currently under treatment or unable to communicate due to severe cognitive or physical impairment were excluded.

Study tools

Sociodemographic variables (age, sex, class, ethnicity, family type, and parental education and occupation) were gathered using an interviewer-administered questionnaire. Modified B. G. Prasad Socioeconomic Classification Scale (updated 2024) [22], which classifies families into five socioeconomic classes (Class I-V) on the basis of monthly per-capita income adjusted to the Consumer Price Index for Industrial Workers, was used for assessment of socioeconomic status. For quantification of mental health status, the DASS-21 scale [19], a validated 21-item scale comprising seven-item subscales measuring depression, anxiety, and stress over the preceding week, was used. Every item is scored on a four-point Likert scale. Severity classification for depression is as follows: Normal 0-9, Mild 10-13, Moderate 14-20, Severe 21-27, and Extremely Severe >28. For anxiety: Normal 0-7, Mild 8-9, Moderate 10-14, Severe 15-19, and Extremely Severe >20. For stress: Normal 0-14, Mild 15-18, Moderate 19-25, Severe 26-33, and Extremely severe >34 [18]. This scale has a high internal consistency (Cronbach’s alpha >0.90) and has been validated for use among Indian adolescents [17,20]. For our study, the scale demonstrated a Cronbach’s alpha of 0.93 for internal consistency.

The Questionnaire was designed in English, with separate sections dedicated to sociodemographic profiles and DASS-21 items. This questionnaire was then translated into simple Hindi by a bilingual investigator and was reviewed by peer investigators. To maintain comprehension, the tool was pretested on a sample of 18 adolescents who were not included in the final analysis. The interviewer administered the questionnaire individually to each participant in a private setting to prevent social desirability bias. Semantic equivalence was confirmed through back-translation. Data quality was ensured by back-checking 10% of completed questionnaires.

Statistical analysis

Data collected using the questionnaire were entered into Microsoft Excel (Microsoft Corporation, Redmond, WA) and analyzed using Jamovi (version 2.6 [Computer Software]. Retrieved from https://www.jamovi.org) software. Summary of categorical variables was presented as percentages and counts. Continuous variables were expressed as mean ± standard deviation for normally distributed variables and median (interquartile range (IQR)) for skewed variables.

To test the normality of continuous DASS-21 scale scores, the Shapiro-Wilk test was used. Groups were compared using a chi-square test (or Fisher's exact test where cell counts were below five) for categorical variables. The Mann-Whitney U test was used to compare nonnormally distributed variables, with rank-biserial correlation (r) reported as effect size. For normally distributed continuous variables, an independent t-test was used, with effect size measure as Cohen’s d.

Prevalence of depression, anxiety, and stress was defined as scoring in a mild or above severity category on the respective DASS-21 subscale. Crude odds ratios (ORs) with 95% CIs were estimated from 2 × 2 contingency tables to quantify the unadjusted association between ethnicity and the mental health outcome.

Only completed questionnaires were analyzed in this study. Of the 340 interviewed adolescents, 30 (8.8%) questionnaires were incomplete and unsuitable for analysis and were discarded during data cleaning. Questionnaire responses were deemed incomplete if more than three DASS-21 items were unanswered or sociodemographic data was missing. The missingness was primarily due to mid-interview participant fatigue or loss of interest during administration. No replacement households were resampled. Field monitoring suggested that incomplete responses occurred randomly across the subgroups. As baseline data were missing for the discarded sample, formal hypothesis testing comparing excluded and included samples was not performed. For all significant statistical tests, p < 0.05 was considered.

## Results

Sociodemographic profile of study participants

The study included 310 adolescents, of whom 173 (55.8%) were nontribal, and 137 (44.2%) were tribal. The majority of participants (45.8%) belonged to the 14-16 years age group, followed by 10-13 years (33.5%) and 17-19 years (20.6%). A significant difference in age distribution between tribal and nontribal groups (χ² = 2.00, p = 0.369) was absent. Girls constituted 59.7% of the sample, and no significant gender difference between the ethnicities (χ² = 0.57, p = 0.450) was observed.

Significant disparities were observed in parental education levels. Among tribal adolescents, 65.0% had fathers with primary/middle school education or less, compared with 32.9% among nontribal adolescents (χ² = 34.8, p < 0.001). Similarly, 62.8% of tribal adolescents had mothers with primary/middle school education or less, compared to 43.4% of nontribal adolescents (χ² = 13.4, p = 0.009). These differences highlight substantial educational disadvantages among tribal families.

Socioeconomic status, assessed using the Modified B.G. Prasad Scale 2024, showed that 46.2% of nontribal adolescents belonged to the high socioeconomic category (Class I + II), compared with 33.6% of tribal adolescents, though this difference was not statistically significant (χ² = 5.28, p = 0.071). Nuclear family structure was predominant in both groups (62.6%), with no significant difference (p = 0.831). The detailed sociodemographic characteristics are presented in Table 1.

**Table 1:** Distribution of demographic characteristics among tribal and nontribal adolescents (n = 310) Sociodemographic characteristics of study participants categorized by ethnicity (nontribal and tribal groups), including age group, gender, father’s educational status, mother’s educational status, socioeconomic status, and family type. Statistical significance was tested using chi-square analysis (χ²). Data are presented as frequencies (n) and percentages (%), calculated columnwise ^*^p < 0.05 is considered statistically significant

Variable	Category	Nontribal, n (%)	Tribal, n (%)	Total, n (%)	χ²	p value
Age group	10-13 years	54 (31.2%)	50 (36.5%)	104 (33.5%)	2.00	0.369
	14-16 years	83 (48.0%)	59 (43.1%)	142 (45.8%)		
	17-19 years	36 (20.8%)	28 (20.4%)	64 (20.6%)		
Gender	Boys	73 (42.2%)	52 (38.0%)	125 (40.3%)	0.57	0.450
	Girls	100 (57.8%)	85 (62.0%)	185 (59.7%)		
Father’s education	Primary/middle or less	57 (32.9%)	89 (65.0%)	146 (47.1%)	34.8	<0.001^*^
	High school/intermediate	39 (22.5%)	25 (18.2%)	64 (20.6%)		
	Graduate	55 (31.8%)	20 (14.5%)	75 (24.2%)		
	Postgraduate	22 (12.7%)	3 (2.2%)	25 (8.1%)		
Mother’s education	Primary/middle or less	75 (43.4%)	86 (62.8%)	161 (51.9%)	13.4	0.009^*^
	High school/intermediate	40 (23.1%)	26 (19.0%)	66 (21.3%)		
	Graduate	47 (27.2%)	22 (16.1%)	69 (22.2%)		
	Postgraduate	11 (6.4%)	3 (2.2%)	14 (4.5%)		
Socioeconomic status	High (Class I + II)	80 (46.2%)	46 (33.6%)	126 (40.6%)	5.28	0.071
	Middle (Class III)	48 (27.7%)	46 (33.6%)	94 (30.3%)		
	Low (Class IV + V)	45 (26.0%)	45 (32.8%)	90 (29.0%)		
Family type	Nuclear	109 (63.0%)	85 (62.0%)	194 (62.6%)	0.05	0.831
	Joint	64 (37.0%)	52 (38.0%)	116 (37.4%)		

Prevalence of depression, anxiety, and stress

The overall prevalence of depression symptoms (any severity above normal) was 63.5% (197/310). Tribal adolescents had significantly higher depression prevalence compared with nontribal adolescents (71.5% vs. 57.2%, χ² = 6.76, p = 0.009, OR = 1.88, 95% CI: 1.16-3.03). This represents nearly two-fold higher odds of depression among tribal adolescents.

Anxiety symptoms were highly prevalent overall (79.0%, 245/310), with tribal adolescents showing a higher prevalence (83.2%) compared to nontribal adolescents (75.7%); however, it was not statistically significant (χ² = 2.59, p = 0.108). Stress symptoms were present in 63.5% (197/310) of adolescents overall. Tribal adolescents had a higher prevalence of stress (69.3%) than nontribal adolescents (59.0%), but the difference was not statistically significant (χ² = 3.56, p = 0.059). Notably, depression was the only domain where a statistically significant ethnic difference in prevalence was observed. The detailed prevalence data are presented in Table 2.

**Table 2:** Prevalence of depression, anxiety, and stress among tribal and nontribal adolescents (n = 310) Prevalence of psychological distress domains (depression, anxiety, and stress) assessed using the DASS-21 scale among adolescents categorized by ethnicity (nontribal and tribal groups). Statistical significance was evaluated using chi-square analysis (χ^2^), with associations presented as ORs and 95% CIs. Data are presented as frequencies (n) and percentages (%), calculated columnwise ^*^p < 0.05 is considered statistically significant OR: odds ratio; CI: confidence interval; DASS-21: Depression, Anxiety, and Stress Scale-21

DASS-21 domain	Nontribal, n (%) (n = 173)	Tribal, n (%) (n = 137)	Total, n (%) (n = 310)	χ²	p value	OR (95% CI)
Depression positive	99 (57.2%)	98 (71.5%)	197 (63.5%)	6.76	0.009^*^	1.88 (1.16-3.03)
Anxiety positive	131 (75.7%)	114 (83.2%)	245 (79.0%)	2.59	0.108	1.58 (0.90-2.77)
Stress positive	102 (59.0%)	95 (69.3%)	197 (63.5%)	3.56	0.059	1.58 (0.98-2.54)

Severity distribution of depression, anxiety, and stress

The severity distribution of depression differed significantly between tribal and nontribal adolescents (χ² = 14.7, p = 0.005). Among tribal adolescents, 28.5% had normal depression scores compared with 42.8% of nontribal adolescents. Moderate depression was present in 29.2% of tribal adolescents vs. 18.5% of nontribal adolescents. Severe depression affected 19.0% of tribal adolescents compared with 9.2% of nontribal adolescents. When combining moderate-to-extremely severe categories, 59.1% of tribal adolescents fell into these higher severity ranges compared with 39.8% of nontribal adolescents, indicating a substantially greater burden of clinically significant depression among tribal youth.

Anxiety severity did not vary significantly between the ethnicities (χ² = 4.99, p = 0.288). However, extremely severe anxiety was notably prevalent in both groups, affecting 42.3% of tribal adolescents and 32.4% of nontribal adolescents. Only 16.8% of tribal adolescents and 24.3% of nontribal adolescents had normal anxiety scores.

The stress severity distribution also did not differ significantly between the groups (χ² = 5.75, p = 0.218). Moderate stress was more common among tribal adolescents (27.7%) compared with nontribal adolescents (18.5%). Normal stress scores were observed in 30.7% of tribal adolescents vs. 41.0% of nontribal adolescents. The detailed severity distributions are presented in Table 3.

**Table 3:** Severity distribution of depression, anxiety, and stress among tribal and nontribal adolescents (n = 310) Severity levels (normal, mild, moderate, severe, and extremely severe) of psychological distress domains (depression, anxiety, and stress) measured using the DASS-21 scale among study participants categorized by ethnicity (nontribal and tribal groups). Statistical significance was evaluated using chi-square analysis (χ^2^). Data are presented as frequencies (n) and percentages (%), calculated columnwise ^*^p <0.05 is considered statistically significant DASS-21: Depression, Anxiety, and Stress Scale-21

DASS-21 domain	Severity category	Nontribal, n (%) (n = 173)	Tribal, n (%) (n = 137)	Total, n (%) (n = 310)	χ²	p value
Depression	Normal	74 (42.8%)	39 (28.5%)	113 (36.5%)	14.7	0.005^*^
	Mild	30 (17.3%)	17 (12.4%)	47 (15.2%)		
	Moderate	32 (18.5%)	40 (29.2%)	72 (23.2%)		
	Severe	16 (9.2%)	26 (19.0%)	42 (13.5%)		
	Extremely severe	21 (12.1%)	15 (10.9%)	36 (11.6%)		
Anxiety	Normal	42 (24.3%)	23 (16.8%)	65 (21.0%)	4.99	0.288
	Mild	11 (6.4%)	6 (4.4%)	17 (5.5%)		
	Moderate	26 (15.0%)	18 (13.1%)	44 (14.2%)		
	Severe	38 (22.0%)	32 (23.4%)	70 (22.6%)		
	Extremely severe	56 (32.4%)	58 (42.3%)	114 (36.8%)		
Stress	Normal	71 (41.0%)	42 (30.7%)	113 (36.5%)	5.75	0.218
	Mild	22 (12.7%)	17 (12.4%)	39 (12.6%)		
	Moderate	32 (18.5%)	38 (27.7%)	70 (22.6%)		
	Severe	29 (16.8%)	24 (17.5%)	53 (17.1%)		
	Extremely severe	19 (11.0%)	16 (11.7%)	35 (11.3%)		

Mean DASS-21 scores

Mean depression scores were significantly higher among tribal adolescents (15.5 ± 9.27) than nontribal adolescents (12.8 ± 9.61), with an independent t-test showing statistical significance (t = -2.48, p = 0.014). The median depression score was also higher among tribal adolescents (16, IQR = 14) than nontribal adolescents (10, IQR = 14). Mann-Whitney U test confirmed this difference (U = 9697, p = 0.006), with a rank-biserial correlation of r = 0.182 and Cohen’s d = 0.28, indicating a small-to-medium effect size. The data for depression scores violated normality (Shapiro-Wilk p < 0.001), and the Mann-Whitney U test was primarily reported.

Mean anxiety scores were higher among tribal adolescents (17.2 ± 9.82) than nontribal adolescents (15.4 ± 10.48), but this difference did not reach statistical significance (t = -1.57, p = 0.118; U = 10,439, p = 0.071), with a small effect size rank-biserial r = 0.119. The data for anxiety scores violated normality (Shapiro-Wilk p < 0.001); the Mann-Whitney U test was primarily reported.

Mean stress scores were also higher among tribal adolescents (20.2±10.70) than nontribal adolescents (18.3 ± 10.90), but again this difference was not statistically significant (t = -1.57, p = 0.117; U = 10,616, p = 0.115), with a small effect size rank-biserial r = 0.104. The data for stress score violated normality (Shapiro-Wilk p < 0.001); therefore, the Mann-Whitney U test was primarily reported. The mean DASS-21 scores are presented in Table 4.

**Table 4:** Mean DASS-21 scores among tribal and nontribal adolescents (n = 310) Comparison of mean scores across psychological distress domains (depression, anxiety, and stress) assessed using the DASS-21 scale between study participants categorized by ethnicity (nontribal and tribal groups). Statistical differences were evaluated using the Independent Samples t-test and Mann-Whitney U test. Data are presented as mean ± SD ^*^p < 0.05 is considered statistically significant SD: standard deviation; DASS-21: Depression, Anxiety, and Stress Scale-21

DASS-21 domain	Nontribal (n = 173) (mean ± SD)	Tribal (n = 137) (mean ± SD)	Mann-Whitney U	p value	Rank-biserial correlation (r)
Depression	12.8 ± 9.61	15.5 ± 9.27	9,697	0.006^*^	0.182
Anxiety	15.4 ± 10.48	17.2 ± 9.82	10,439	0.071	0.119
Stress	18.3 ± 10.90	20.2 ± 10.70	10,616	0.115	0.104

Overall mental health burden

The overall mental health burden across both groups is as follows. Tribal adolescents consistently showed a higher burden across all three DASS-21 domains. Depression was the only domain demonstrating statistically significant ethnic differences in both prevalence and severity. Overall, 63.5% of all 310 adolescents screened positive for at least depression or stress symptoms, indicating a substantial mental health burden in this rural population.

## Discussion

Findings of this study indicate a high burden of mental health symptoms among adolescents in Jharkhand, with tribal adolescents facing significantly higher risk of depression. Our finding of a 63% overall prevalence of depression exceeds that reported in previous studies; for example, Hebbani et al. reported a 35.5% prevalence in South India [23] and a 45.3% prevalence in West Bengal, as reported by Gupta et al. [6]. The socioeconomic constitution of our rural site and tribal population exposed to structural disadvantages and limited access to mental health services may explain this disparity. The difference in findings may also be explained by the sensitivity of the DASS-21 scale, which, being a screening tool, aggregates subclinical manifestations that standard diagnostic interviews often exclude [4,5]. Thus, prevalence estimates obtained using DASS-21 are expected to exceed those based on structured psychiatric interviews. This study was done in a rural setting with a sizable tribal population and is characterized by socioeconomic disadvantage, lower parental education, and scarce mental health services; these factors may be responsible for poor mental health outcomes. The sample population of this study was recruited from the community rather than schools; this allowed for the inclusion of adolescents who might otherwise be absent from school due to socioeconomic hardship, illness, or other vulnerabilities that increase psychological distress.

Our findings align with an emerging body of Indian literature documenting mental health disparities among tribal populations in India. Verma et al. [24] reported elevated mental health morbidities among tribal populations. Suting and Ali [25] identified risk and protective factors for these morbidities in the tribal population of Meghalaya; Shrivastav et al. [26] documented gender-based disadvantage among tribal youth in Central India. Our study extends this existing literature by providing quantitative estimates of prevalence and comparative analysis in a rural block of Jharkhand. Our findings suggest comparatively poorer socioeconomic conditions and lower parental education attainment among tribal adolescents compared with their nontribal counterparts. Culminating in historical social disadvantage, tribal adolescents in this study suffer from a greater burden of depressive symptoms compared with their nontribal counterparts. Levels of anxiety and stress remained high among both groups, showing no statistically significant disparity.

The deficit of mental health infrastructure in rural areas possibly elevated the psychological impact of the COVID-19 pandemic; this is supported by studies conducted during and after the COVID-19 pandemic that reported elevations in anxiety symptoms among adolescents [8,9]. Our observations support this, as observed overall anxiety burden (79% overall) in this study is substantially higher than historical prepandemic estimates from Indian studies [27]; however, prevalence did not differ significantly between tribal and nontribal groups. Since specific COVID-19 exposure variables were not directly measured in this study, the postpandemic impacts on mental health remain context-dependent and should be interpreted with caution.

Parental education level is associated with reduced health literacy, constrained economic opportunities, limited awareness of mental health issues, and decreased ability to navigate health systems. In our study, 65% of tribal adolescents had fathers with primary/middle school education or less compared to 32.9% of nontribal adolescents (p < 0.001). This finding resonates with recent studies that documented socioeconomic inequalities as fundamental determinants of adolescent mental health [28].

The threshold used in this study defined any person with mild or greater symptom severity as screening positive. This inevitably increases prevalence estimates compared with studies reporting only moderate or clinically significant disorders. Findings of the present study should be interpreted as reflecting the burden of mental health symptoms requiring further assessment rather than the prevalence of clinically diagnosed disorders.

Limitations

Limitations of this study also need to be acknowledged. First, the DASS-21 is a screening instrument that detects symptoms rather than providing clinical diagnoses; adolescents screening positive require further evaluation by mental health specialists. Second, the study was conducted in a single block in Ranchi, Jharkhand, limiting its generalizability to other populations or geographical regions. Third, self-report measures are subject to recall bias and social desirability bias, though the use of a validated instrument and confidential data collection procedures minimize these concerns. Fourth, purposive sampling of households enrolled under the RHTC may introduce selection bias, though consecutive sampling of all eligible adolescents within enrolled households reduces this concern. However, rural adolescents who attend boarding/residential schools, work outside home during daytime, or have temporarily migrated may have been systematically excluded or underrepresented in our study. This could potentially underestimate psychological distress among this excluded cohort of adolescents. Fifth, our study relied on bivariate statistical tests and crude ORs to assess associations. The absence of multivariable logistic regression limits the ability to confirm ethnicity as an independent risk factor for depression or to determine whether the observed associations are confounded by other factors, such as socioeconomic disparities and differences in parental education. Finally, causality cannot be determined using a cross-sectional design; longitudinal studies are needed to establish temporal relationships between risk factors and mental health outcomes.

## Conclusions

This study documents a high burden of mental health symptoms in rural adolescents in Jharkhand, with 63.5% screening affirmative for depression, 79.0% for anxiety, and 63.5% for stress. Tribal adolescents bear a significantly higher depression burden (OR = 1.88, p=0.009), with 59.1% experiencing moderate-to-extremely severe depression compared with 39.8% of nontribal adolescents. While statistically significant ethnic disparity was observed in the tribal adolescents specifically for depression compared to nontribal adolescents, symptom burdens for anxiety and stress were elevated across both groups without significant intergroup differences.

Structural factors like lower socioeconomic disparity and parental education were univariately associated with higher mental health distress and represent important potential correlates. Multisectoral interventions like addressing educational disparities and community counseling might help mitigate adolescent mental health distress in rural areas. Interventions should include the establishment of culturally sensitive community-based counseling, introducing school-based screening of mental health issues like stress, anxiety, and depression, and addressing structural inequalities.

The growing burden of mental health issues among adolescents represents both a public health crisis and an opportunity to intervene. This study provides baseline evidence regarding the mental health status of adolescents in a rural block of Ranchi, Jharkhand, and can be helpful in designing evidence-based, targeted mental health interventions. Early identification and treatment of adolescent mental health issues have the potential to reduce suicide risk, improve educational outcomes, enhance occupational productivity, and are instrumental in preventing progression to chronic disorders.
